# Hypnotism

**Published:** 1909-06

**Authors:** 


**Hypnotism.** By Dr. August Forel. Translated from the Fifth German Edition by H. W. Armit. Pp. xii, 370. London: Rebman Limited. 1906.

This, the fifth edition of Dr. Forel's well-known work on Hypnotism, contains certain interesting and important additions to the theoretical phase of this subject. Considerable attention is given to the exposition of his theory of Monistic Identity; that is, the unity of each physiological phenomenon, and its corresponding cortical molecular activity. Mind and body are thus but two aspects of the same thing. That his hypothesis does not harmonise with the Law of Fechner-Weber he ascribes to the interposition of complicated brain centres and their hypo-conceived activity, in its nature facilitating, disturbing, or inhibitory. The author deals subsequently very lucidly with the different planes of consciousness, alternate and serial double consciousness, and states of automatism. He shows how, in dissociated states of brain, hypoconscious associated connections acting vividly may be found, and enchain with them the ego, or may even divide it into a double ego, or duplex personality. Semon's theory of the Mneme or Organic Memory is freely quoted and reviewed. It is considered possible that slow inheritance of acquired characteristics may result from the long continued ecphorisation of engrams, which radiate into the entire organism, and are capable of affecting embryonic cells. Semon's views are again discussed in connection with so-called " Negative " Hallucinations. What he terms Alternative

Euphoria, both simultaneous and successive, affords explanation of these, as also of the possibility of displacing actual sense-stimulation by suggestive ecphorisation. Dealing with the subject of Suggestion, the author emphasises that normally all are intuitively credulous or suggestible. Belief and intuition are complexes largely occurring below the level of superconsciousness: thus Suggestion can take place hypoconsciously, and, in fact, the more the exercise of the superconscious will, the less the suggestibility. The endeavour to assume passivity, as well as mental agitation and resistive attitude, render hypnosis difficult or impracticable. The importance of contrasting impressions (which normally are especially active where the attempt is made to arouse subjective states) is dwelt upon in connection with auto-suggestion, as explaining faulty results in hypnotisation; also complementary and opposite states in so-called negative and positive hallucinations in relation to somnambulistic and insane conditions.

The mechanism of natural sleep and the hypnotic state are the same: both are states of dissociation of consciousness, are states of heightened suggestibility. In sleep, however, the element of exhaustion is added. The criteria of hypnotic consciousness are those of the dream existence, hallucinations of perceptions, exaggeration of feelings and their motor reflexes, dissociation of organic local associations of engram complexes. Forel regards hysteria as a dissociative weakness of the brain, by which a pathological auto-suggestibility is caused: an instability in which conceptions, impulses of will and emotions, are with facility disintegrated. The reaction to Suggestion is morbid, unintentional auto-suggestions are added, there is wholesale production of unprompted paralyses, convulsions, pains, &c. This pathological auto-suggestibility is more deeply morbid in kind than the phenomenon of hetero-suggestibility. Hysteria as a constitutional malady is incurable. The symptoms are ameliorable, not the disposition. That form produced by exhaustion (neurasthenia), or peripheral irritation, is, however, curable.

Some causes of failure in producing hypnosis have been alluded to, principally antagonistic auto-suggestion. The insane are largely unhypnotisable, by reason of the permanent morbid irritation of their brains; or, again, diseased inhibitions or conditions of stimulation underlying delusions, too intense to be dissociated. It is much easier to influence a definite local phenomenon, such as alcoholic craving, than general dispositions. Acquired habits, too, may be removed, but constitutional characteristics are doubtfully to be influenced, and certainly not permanently. Forel here emphasises the need of complete and permanent abstinence from the enslaving drug or habit after cure has been effected; also that the methods of treatment for all neuropathic cases should be carefully adjusted to each, and intelligent occupations—not mere mechanical routine—should be found.

The influence exerted in hypnosis may extend itself post-hypnotically into the normal condition of mind, and may include that of the operator ; but the reaction to post-hypnotic suggestion will probably depend upon the relative suggestibility of the individual, his normal standard of morality and control, his hypnotic training, and the depth of hypnosis. The higher degrees of somnambulism and certain hysterical persons are deeply and entirely affected by suggestion, and Forel is convinced that any conceivable crime may be committed in the person of one hypnotised, provided that a high degree of hypnosis be obtained. There is safety, however, in the guardedness of the patient, in the sudden and unexpected returns to consciousness to which he is liable, and to the possibility of future revelation under subsequent hypnosis by others. Forel is resolute in the denial of any serious or lasting damage, mental or bodily, which can be occasioned by hypnotism when properly applied. The translator's work is admirably done, and the book is to be unreservedly commended.

